# A fourth dose of the inactivated SARS-CoV-2 vaccine redistributes humoral immunity to the N-terminal domain

**DOI:** 10.1038/s41467-022-34633-7

**Published:** 2022-11-11

**Authors:** Ji Wang, Caiguangxi Deng, Ming Liu, Yihao Liu, Liubing Li, Zhangping Huang, Liru Shang, Juan Jiang, Yongyong Li, Ruohui Mo, Hui Zhang, Min Liu, Sui Peng, Haipeng Xiao

**Affiliations:** 1 Department of Endocrinology, The First Affiliated Hospital, Sun Yat-sen University, 510080 Guangzhou, People’s Republic of China; 2 Institute of Precision Medicine, The First Affiliated Hospital, Sun Yat-sen University, 510080 Guangzhou, People’s Republic of China; 3 Clinical Trials Unit, The First Affiliated Hospital, Sun Yat-sen University, 510080 Guangzhou, People’s Republic of China; 4 Department of Laboratory Medicine, The First Affiliated Hospital, Sun Yat-sen University, 510080 Guangzhou, People’s Republic of China; 5 Department of Rheumatology and Clinical Immunology, The First Affiliated Hospital, Sun Yat-sen University, 510080 Guangzhou, People’s Republic of China

**Keywords:** Immunology, Medical research, SARS-CoV-2, Inactivated vaccines

## Abstract

The effectiveness of a 3^rd^ dose of SARS-CoV-2 vaccines waned quickly in the Omicron-predominant period. In response to fast-waning immunity and the threat of Omicron variant of concern (VOC) to healthcare workers (HCWs), we conduct a non-randomized trial (ChiCTR2200055564) in which 38 HCWs volunteer to receive a homologous booster of inactivated vaccines (BBIBP-CorV) 6 months after the 3^rd^ dose. The primary and secondary outcomes are neutralizing antibodies (NAbs) and the receptor-binding domain (RBD)-directed antibodies, respectively. The 4^th^ dose recalls waned immunity while having distinct effects on humoral responses to different antigens. The peak antibody response to the RBD induced by the 4^th^ dose is inferior to that after the 3^rd^ dose, whereas responses to the N-terminal domain (NTD) of spike protein are further strengthened significantly. Accordingly, the 4^th^ dose further elevates the peak level of NAbs against ancestral SARS-CoV-2 and Omicron BA.2, but not BA.1 which has more NTD mutations. No severe adverse events related to vaccination are recorded during the trial. Here, we show that redistribution of immune focus after repeated vaccinations may modulate cross-protective immune responses against different VOCs.

## Abstract (web summary)

Despite the availability of booster doses against SARS-CoV-2, variants of concern are capable of escaping immune responses established by vaccination or natural infection. In this work, the authors detail the immune responses elicited in a cohort of healthcare workers, receiving a fourth dose of inactivated vaccine.

## Introduction

Vaccination is one of the most cost-effective ways to prevent infectious diseases, including COVID-19. Billions of vaccine doses have been distributed worldwide and showed promising effectiveness against SARS-CoV-2 infection and related hospitalization. However, the vaccine-induced immune response waned rapidly after receiving two doses of mRNA vaccines^1^. Our previous study also showed humoral immune responses elicited by inactivated SARS-CoV-2 vaccines declined quickly within 6 months after a standard two-dose vaccination regimen^2^. In addition to the fast-fading immune response, the frequent emergence of mutated SARS-CoV-2 viruses, especially those variants of concern (VOC), further challenges the vaccination system based on the ancestral viral strain^3,4^.

Therefore, a booster or a 3rd dose of vaccines was provided globally. Our previous study and others have demonstrated that the 3rd dose elevated both humoral and cellular immune responses to a much greater level than the two-dose regimen, equipping the population with potent protection not only for the ancestral virus but also for VOCs, such as the Delta variant^2,5^. Unfortunately, recently emerged VOC Omicron carries more than 30 mutations, rendering an overwhelming capability of escaping immune responses established by vaccination or natural infection^4^. Numerous breakthrough infections have been reported worldwide^6^. BA.1 was the first Omicron lineage that spread globally, and a more transmissible lineage BA.2 subsequently overtook BA.1 to become the predominant VOCs at the moment^7^. Most recently, several BA.2-derived new Omicron sub-lineage raised new concerns. For instance, more than one thousand confirmed infections by Omicron XE, a BA.1/BA.2 recombinant with a 12.6% higher growth rate over BA.2, has been reported worldwide^8^. Moreover, BA.4 and BA.5 with two more mutations (L452R and F486V) in the receptor binding domain (RBD) could escape BA.1 infection elicited neutralizing immunity, and are sweeping the world^9^.

While antibodies induced by the 3rd dose of vaccines do neutralize Omicron to some extent and T cell responses are cross-reactive, preliminary data have shown that the protection against viral infection provided by the booster dose was not complete and also waned at a fast pace^10–12^. A recent report from US CDC revealed that the vaccine efficiency against the emergency department and urgent care encounters in people who had received 3 doses of mRNA vaccines declined from 87% to 66% within 4 months, and further dropped to 31% after 5 months in the Omicron-predominant period^10^. Thus, in early January, Israel began to provide a 4th dose of vaccines to the most vulnerable populations, including Healthcare workers (HCWs)^13,14^.

In this work, we find only 15% of humoral immune responses remain 6 months after receiving three doses of inactivated SARS-CoV-2 vaccines. In response to the fast-waning immune responses and a great threat of Omicron to the healthcare system, 38 HCWs who were in our previous cohort investigating responses to the first three doses of inactivated vaccines participate in the current study and receive a 4th homologous booster. The immune responses against both ancestral SARS-CoV-2 strain and Omicron variant are monitored along with a longitudinal assessment of humoral responses to multiple antigens and domains. After the 4th dose, a stepwise elevation of peak humoral immune responses following the first three doses does not continue for most antigens or domains, whereas a further enhanced N-terminal domain-directed immune response is observed. The 4th dose does not further enhance the peak neutralizing antibody (NAbs) response against Omicron BA.1 variant, but encouragingly the peak level of NAbs against BA.2 is significantly elevated.

## Results

### The immune response induced by the 3rd dose of vaccines waned rapidly

We have previously conducted a non-randomized trial and recruited HCWs from a prospective cohort. They received a primary 2-dose series of the inactivated SARS-CoV-2 vaccine (BBIBP-CorV, Sinopharm, Beijing) in January and February 2021, followed by a 3rd dose booster of the same vaccine 5 months later in July 2021^2,15^. Since Omicron had been threatening the healthcare systems by the end of 2021, Thirty-eight HCWs from the previous cohort volunteered in the current non-randomized trial (ChiCTR2200055564) to investigate the longevity of immune responses after the 3rd dose and the potential benefit of a 4th dose.

**Fig. 1:**
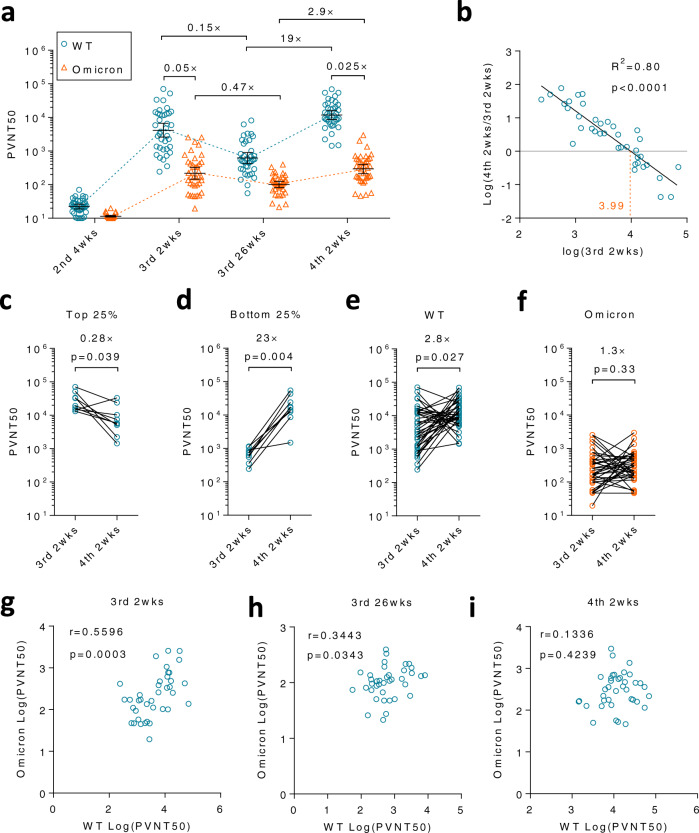
A 4th dose of inactivated SARS-CoV-2 vaccine boosted NAbs against wildtype (WT) virus but not Omicron BA.1. Thirty-eight HCWs who have already received 3 doses of inactivated SARS-CoV-2 vaccine volunteered in this clinical study and received a 4th homologous dose 6 months (26 wks) after the 3rd dose. **a** Neutralization assays were performed to measure NAbs titers against pseudoviruses with S protein from a WT strain (blue circle) or Omicron BA.1 variant (orange triangle). **b** Linear regression was performed on the fold-change of half pseudovirus neutralization titers (PVNT50) from 3rd 2 wks to 4th 2 wks and PVNT50 at 3rd 2 wks. The orange dashed line is a calculated threshold. The peak NAb levels could be further elevated by the 4th dose if the peak level after the 3rd dose is below this threshold. **c** The change of NAbs was shown for participants with the top 25% highest PVNT50 against WT virus. **d** The change of NAbs was shown for participants with the lowest (bottom 25%) PVNT50 against WT virus. NAbs at 3rd 2 wks and 4th 2 wks against WT virus (**e**) or Omicron variant (**f**) were compared respectively. The two-tailed spearman correlation coefficient was calculated for PVNT50 to WT and Omicron at 3rd 2 wks (**g**), 3rd 26 wks (**h**), or 4th 2 wks (**i**). For (**a**, **b**, **e**–**i**), *n* = 38 biologically independent samples. For (**c**, **d**), *n* = 9 biologically independent samples. Data were shown as Geometric mean ± 95% confidence level (Cl). The two-tailed spearman correlation coefficient was used for (**b**). Two-tailed Wilcoxon matched-pairs signed rank test was used for (**c**–**f**). Source data are provided as a Source Data file.

Serum neutralizing antibodies (NAbs) against an ancestral SARS-CoV-2 viral strain (Wuhan-Hu-1), named wildtype (WT) hereafter, or the Omicron variant BA.1 lineage (Omicron) were measured by a pseudovirus assay and quantified as half pseudovirus neutralization titers (PVNT50)^16^. The geometric mean of neutralization titers (GMNT) against WT drastically decreased by 85% 26 weeks (wks) after the 3rd dose, as compared to GMNT at 2 wks after the 3rd dose (Fig. 1a, blue circles in 1st and 2nd panels). As expected, GMNT against Omicron was much lower than that against WT at these two time points, though the immune response against Omicron seemed to drop at a slower pace (Fig. 1a, orange triangles in 1st and 2nd panels). GMNT against Omicron decreased by 53% within 6 months. No significant difference was observed between male and female HCWs at any time point (Supplementary Fig. 1a, b).

### The 4th dose of vaccination recalled the waned immune response

These HCWs received the 4th dose of the inactivated SARS-CoV-2 vaccine (BBIBP-CorV, Sinopharm, Beijing) in January 2022. No severe side effects related to vaccination were recorded during the trial (Table 1). The 4th dose robustly recalled NAbs titers against WT by 19 (11.6, 31.28) folds (Fig. 1a, blue circles in 3rd panel). Cross-reactive NAbs to Omicron were successfully also recalled despite to a lesser extent, 2.9 (2, 4.1) folds (Fig. 1a, orange triangles in 3rd panel).

**Table 1:** Demographics and vaccination-related adverse events

Participants, *N*		38
Age, mean (SD)		27.63(6.70)
Sex, male (%)		18 (47.4)
Adverse events, *N* (%)		7(18.4)
Injection site symptoms	Pruritus, *N* (%)	2(5.3)
	Swollen, *N* (%)	1(2.6)
Systematic symptoms	Rash, *N* (%)	1(2.6)
	Dizziness, *N* (%)	2(5.3)
	Nausea, *N* (%)	1(2.6)
	Fatigue, *N* (%)	1(2.6)

### Immunity induced by the 3rd dose affected the outcome of the 4th dose

As SARS-CoV-2 continuously circulates globally waves by waves, multiple vaccinations are needed. Thus, it is of great importance to know the impact of previous vaccination on the following booster. We first analyzed the correlation between NAb titers at various time points, finding that neither titers at 3rd 2 wks nor 3rd 26 wks correlated with titers at 4th 2 wks (Supplementary Fig. 2a, b). Surprisingly, a reverse correlation between NAb titers at 2 wks after the 3rd dose (3rd 2 wks) and the fold change from the 3rd peak to the 4th peak (4th 2 wks/3rd 2 wks) was observed (*R*^2^ = 0.80, *p* < 0.0001), indicating whether or not the peak humoral response of the 4th vaccination could be further elevated depended on the peak of the previous vaccination (Fig. 1b). In contrast, residual immune responses at 3rd 26 wks, the immune responses right before the 4th vaccination, had little impact on the outcome of the 4th dose (Supplementary Fig. 2c). Collectively, these data demonstrated that it is the peak response after the 3rd vaccination that determined whether a breakthrough from the previous peak would take place after the 4th dose.

As a result of this negative correlation, inferior titers at 4th 2 wks to those at 3rd 2 wks were always detected among the top 25% of participants with the highest NAb titers at 3rd 2 wks. GMNT decreased by 3.6 (1.4, 9.7) folds (Fig. 1c). Conversely, the bottom 25% of participants who had a low immune response benefited most from a drastic increase of 23 (9.6, 55.8) folds in NAb titers after the 4th vaccination (Fig. 1d). A similar dramatic increase in peak GMNT from the 2nd to the 3rd dose was also observed in all participants whose NAb titers are relatively low after the 2nd dose (Supplementary Fig. 2d). These results suggest that only those individuals who respond less well to the first 3 doses are preferable for the 4th dose in terms of NAbs against WT virus. The indicator is the peak response after the 3rd dose, whose cut-off value is a PVNT50 of 1 × 10^3.99^ in our pseudovirus system (Fig. 1b, orange dashed line).

### The 4th dose had distinct effects on WT virus and Omicron variant

As mentioned above, the elevation from 3rd 26 wks to 4th 2 wks was much greater for WT than that for Omicron (19-fold vs 2.9-fold) (Fig. 1a). We next compared peak NAb titers after the 3rd dose and the 4th dose. GMNT increased by 2.8 (1.5, 5.4) folds for WT, whereas no significant change was observed for Omicron (Fig. 1e, f). This data indicated that whilst the 4th vaccination further enhanced WT-specific immune responses, the cross-neutralizing immune response was not equally strengthened. As a result, the GMNT ratio between two viral strains dropped from 0.05 to 0.025 after the 4th dose (Fig. 1a, 1st and 3rd panels). To further validate the different effects of the 4th dose on two strains, the spearman correlation coefficient analysis was performed between NAb titers against WT and Omicron at various time points. As expected, a significant albeit moderate correlation (*p* = 0.0003) was seen at 3rd 2 wks, but gradually lost over time and was disrupted (*p* = 0.42) after the 4th dose (Fig. 1g–i). Taken together, these results suggested a shift of immune responses from shared epitopes to non-cross-reactive ones after the 4th vaccination.

### The trajectory of humoral immune responses to multiple antigens

**Fig. 2:**
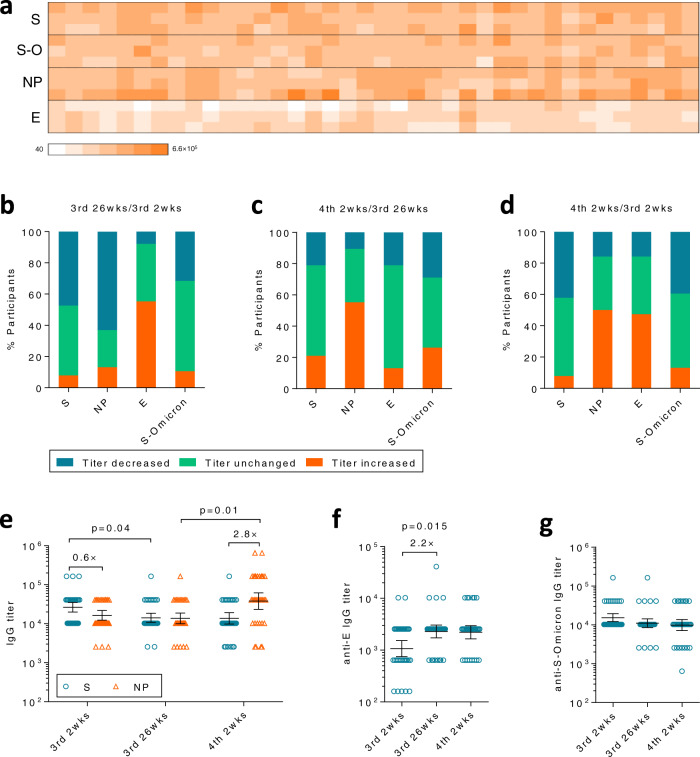
Humoral immune responses against multiple antigens. **a** Anti-spike (S), Nucleocapsid (NP), Envelope (E), and S-Omicron (S-O) antibodies were measured by ELISA at 2 weeks after the 3rd dose (3rd 2 wks), 26 weeks after the 3rd dose (3rd 26wks) or 2 weeks after the 4th dose (4th 2wks). **b**–**d** Antibody titers for each protein were compared between 3rd 2 wks and 26 wks (**b**), 4th 2 wks and 3rd 26 wks (**c**), 4th 2 wks and 3rd 2 wks (**d**), respectively. Percentage of participants with increased titers (former/later >1, orange), decreased titers (former/later <1, blue) or unchanged titers (former/later =1, green) were summarized. **e** Anti-S (blue circle) and anti-NP (orange triangle) antibody titers were compared between each time point. **f** Anti-E antibody titers at each time point were compared. **g** Antibody titers for S-Omicron. For (**e**–**g**), *n* = 38 biologically independent samples. Data were shown as Geometric mean ± 95% Cl. Two-tailed Friedman test followed by Dunn’s multiple comparisons test was used for (**e**–**f**). Source data are provided as a Source Data file.

The inactivated SARS-CoV-2 vaccine contains all viral structure proteins, among which Spike protein (S), Nucleocapsid (NP), and Envelope (E) are immunogenic as revealed by our previous study^2^. Among these immunogenic viral proteins, only S protein plays the pivotal role in inducing NAbs, while the contribution of other proteins may be minimal^17^. Thus, we next investigated whether the 4th dose altered the distribution of humoral immune responses to various antigens (Fig. 2a). As expected, anti-S and anti-NP antibody titers decreased in a large portion of participants between 2 and 26 weeks after the 3rd dose (Fig. 2b, 1st and 2nd bars), except for anti-E antibody titers which increased in >50% of participants in this period (Fig. 2b, 3rd bar). Unexpectedly, the 4th dose did not effectively recall anti-S and anti-E antibodies in a majority of participants, while it did successfully recall anti-NP antibodies in >50% of individuals of the same population (Fig. 2c, 2nd bar).

As a result, most participants had inferior peak anti-S antibody titers after the 4th dose as compared to peak titers after the 3rd dose (Fig. 2d, 1st bar), in sharp contrast to anti-NP antibodies whose peak titers were further elevated by the 4th dose in >50% of individuals (Fig. 2d, 2nd bar). Anti-S antibody titers slightly dropped from 2 wks to 26 wks after the 3rd dose, but did not raised after the 4th dose (Fig. 2e, blue circles). In contrast, anti-NP antibody titers only dropped slightly before the 4th dose but greatly raised thereafter (Fig. 2e, orange triangles). As a result, the ratio of peak antibody titers between anti-NP and anti-S was reversed by the 4th dose. Anti-NP was 40% lower than anti-S right after the 3rd dose, but became 2.8 (1.9, 4.1) folds higher after the 4th dose (Fig. 2e, 1st and 3rd panels).

We further measured cross-reactive antibodies to Omicron Spike protein (S-Omicron), observing a similar decreasing trend as ancestral S protein albeit titers were slightly lower (Fig. 2b–d, g). Distinct trends of anti-NP and anti-S titers suggested that the immune system began to focus on a more immunodominant antigen, such as NP, after repeated vaccination of the inactivated vaccines that comprise multiple viral proteins. Antibodies against E, another immunogenic antigen in the vaccine, showed a slightly increasing trend from the 3rd to the 4th dose (Fig. 2f). However, the impact of this change on anti-S antibodies and the protection remained elusive since anti-E antibody titers were one magnitude lower than that of anti-S or anti-NP antibodies (Fig. 2e, f).

### ADCC and T cell responses against multiple antigens

**Fig. 3:**
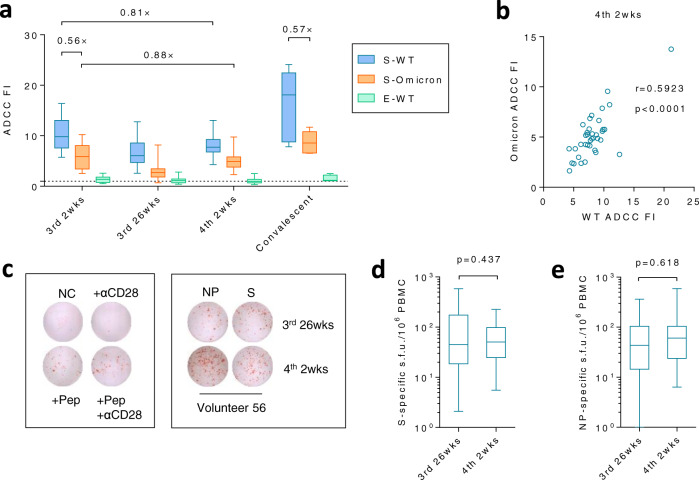
ADCC and T cell responses against multiple antigens. **a** ADCC fold induction (FI) of each sample against unimmunized control was summarized. The dashed line indicates the threshold, sample/unimmunized = 1. Convalescent patients who had recovered from WT SARSCoV-2 infection were recruited as the positive control^2^. **b** The two-tailed spearman correlation coefficient was calculated on ADCC activities against WT and Omicron at 4th 2 wks. **c**–**e** T cell responses after the 3rd and 4th dose were measured by ELISPOT. Controls and representative spots from volunteer 56 were shown in (**c**), while S-specific or NP-specific T cell responses were summarized in (**d**) and (**e**), respectively. Pep, peptide pool. s.f.u., spot-forming units. For (**a**, **b**, **d**, **e**), *n* = 38 biologically independent samples, except for the convalescent patient group in which *n* = 5 biologically independent samples. Data were shown as box and whiskers, indicating median (middle line), 25th, 75th percentile (box) and 5th and 95th percentile (whiskers). Two-tailed Wilcoxon matched-pairs signed rank test was used for (**d**, **e**). Source data are provided as a Source Data file.

Since humoral immune responses against NP or E protein could not mediate neutralization, we next investigated if these antigens could contribute to the protection via inducing other types of immune responses, such as Antibody-dependent cellular cytotoxicity (ADCC) or T cell responses which may contribute to disease prevention^17,18^. Neither vaccine-induced nor infection-induced anti-E antibodies had strong ADCC activities when compared to S-directed antibodies (Fig. 3a). Intriguingly, S-directed antibodies exhibited substantial ADCC activities against both WT and Omicron S-expressing targeted cells, though the level was slightly lower than that of convalescent patients who had recovered from WT SARS-CoV-2 infection^2^. ADCC effects against WT or Omicron were tightly correlated (Fig. 3b). Mutations of Omicron only rendered a moderate loss of ADCC (~2-fold), in sharp contrast to a substantial loss of neutralization (~20-fold), partially explaining why vaccination may still reduce hospitalization in absence of NAbs (Figs. 1a, 3a). The trajectory of ADCC mirrored the trend of S-directed antibodies that the mean titer at 4th 2 wks was inferior to that at 3rd 2 wks (Fig. 2e, g, h). Significant amounts of S- or NP-specific T cell responses were readily detected by ELISPOT 6 months after the 3rd dose albeit no further enhancement was observed after the 4th dose (Fig. 3c–e).

### Impact of the 3rd dose on the induction of RBD-NAbs after the 4th dose

Reduced efficacy of the 4th dose on elevating anti-S antibodies partially explained why the 4th did not dramatically elevate the peak of NAbs as the 3rd dose did (Fig. 1a, 1st and 2nd panels), but did not explain why the NAbs to WT still increased slightly while those to Omicron did not (Fig. 1e, f). Whilst ADCC activities against WT and Omicron were tightly correlated at all time points (Fig. 3b and Supplementary Fig. 3), the correlation on neutralization between WT and Omicron was lost after the 4th dose, indicating that the composition of NAbs against different domains on S protein had been altered by the 4th dose (Fig. 1g–i).

**Fig. 4:**
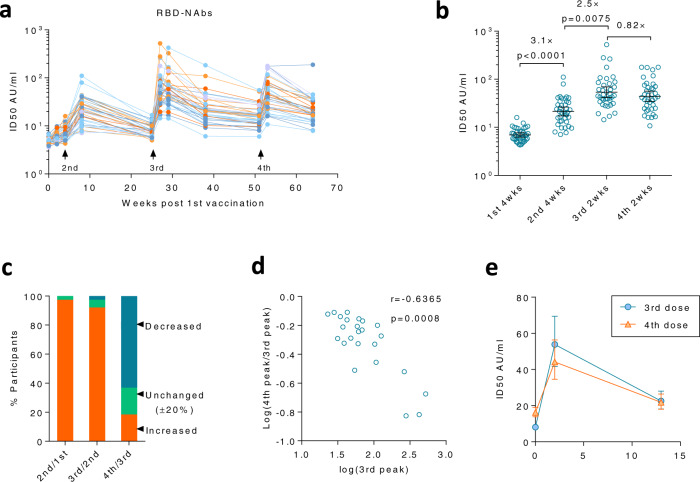
The 4th dose did not further elevate the peak level of RBD-Nabs. **a** NAbs to RBD (RBD-NAbs) were measured by a one-step competitive Chemiluminescent immunoassay for sera collected at various time points after 1st, 2nd, 3rd, and 4th doses. The kinetic of RBD-NAbs was shown for each participant. AU, arbitrary units. **b** Peak values of RBD-NAbs after each vaccination were compared. **c** Percentage of participants with increased peak value (increased >20%, orange), decreased value (decreased >20%, blue), or unchanged (±20%, green) as compared to the previous peak were summarized. **d** The two-tailed spearman correlation coefficient was calculated for the peak value after the 3rd dose and the fold change of peak value from the 3rd to the 4th dose in the decreased group shown in (**c**). **e** Kinetics of RBD-NAbs were compared among various time points after the 3rd and 4th dose. For (**b**, **e**), *n* = 38 biologically independent samples. Data were shown as Geometric mean ± 95% Cl. RM one-way ANOVA followed by Bonferroni’s multiple comparisons test was used for (**b**). Source data are provided as a Source Data file.

As the RBD plays a crucial role in inducing NAbs, we first investigated whether NAbs to RBD were altered. Thus, a one-step competitive Chemiluminescent immunoassay was used to detect the concentration of NAbs that compete with ACE2 binding to RBD^19^. Whilst the 3rd dose induced robust immune responses at 2 and 4 weeks after immunization, the RBD-NAbs waned quickly (Fig. 4a). An average drop of 60% from the peak was observed within 3 months after the 3rd dose (Supplementary Fig. 4a). Although only 25% of immune responses remained after 6 months, residual immune responses were still significantly higher than that at 5 months after the 2nd dose (Supplementary Fig. 4b). The 4th dose effectively elevated RBD-NAbs in all vaccinees, whose geometric mean RBD-NAbs level increased by 2.7 (2.2, 3.3) folds within 2 weeks after immunization as compared to those at 6 months after the 3rd dose (Supplementary Fig. 4a). The 4th dose was equally effective for both females and males in inducing RBD-NAbs (Supplementary Fig. 5). Geometric mean RBD-NAbs remained comparable between 3rd 13 wks and 4th 13 wks (Fig. 4e).

Whilst the first three doses resulted in a stepwise elevation of peak RBD-NAbs, the 4th dose did not. The 2nd and 3rd dose enhanced RBD-NAbs by 3.1 (2.6, 3.7) folds and 2.5 (2, 3.2) folds to the previous peak, respectively (Fig. 4b). However, the 4th dose did not further elevate the peak value of RBD-NAbs, but an 18% decrease was found instead (Fig. 4b). The inferior peak value after the 4th dose did not happen sporadically. In 63% (24/38) of participants, the peak RBD-NAbs after the 4th dose was 20% lower than that after the 3rd dose, whereas only 18% (7/38) increased by >20% (Fig. 4c, 3rd bar). In sharp contrast, >90% of participants (37/38 and 35/38) benefited from each booster shot of the 2nd and the 3rd dose (Fig. 4c, 1st and 2nd bars).

As indicated by our pseudovirus neutralization data, immune responses induced by the previous vaccination may negatively affect NAbs induced by the following dose (Fig. 1b, c). However, the neutralization assay involved antibodies induced by multiple antigens or domains. Here, we explored whether it is true for the immune response to a single domain as well. A significant negative correlation between fold-change of peak values and the values of previous peaks was observed after the 4th dose in participants with decreased peak RBD-NAbs, indicating that potent immune responses elicited by the 3rd dose might suppress the induction of RBD-NAbs after the 4th dose (Fig. 4d).

### The 4th dose redistributed the humoral immune response from RBD to NTD

As most cross-neutralizing antibodies against Omicron BA.1 targeted RBD^4^, a slightly decreased peak RBD-NAbs level could explain why the 4th dose did not further boost the peak NAbs level against Omicron BA.1. On the other hand, peak NAbs levels against WT were still elevated by the 4th dose, indicating that immune responses to other domains of S protein may be increased and compensate for the loss of RBD-NAbs.

**Fig. 5:**
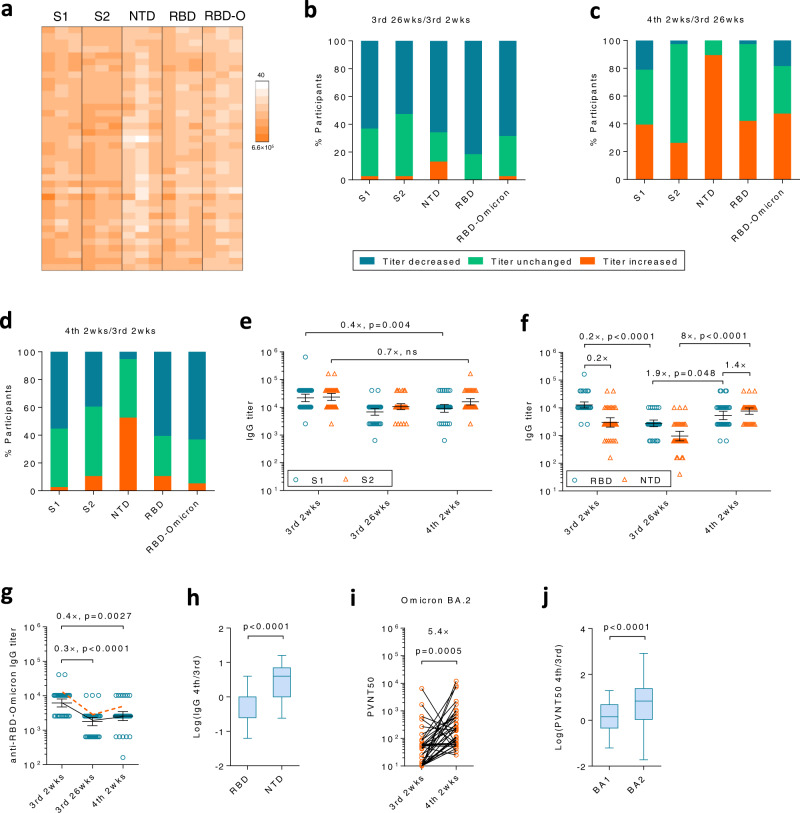
The S protein directed antibody responses shifted from RBD to NTD. **a** Anti-S1 domain, S2 domain, N-terminal domain (NTD), Receptor binding domain (RBD) and RBD-Omicron (RBD-O) antibodies were measured by ELISA at 3rd 2 wks, 3rd 26 wks or 4th 2 wks. **b**–**d** Antibody titers for each protein were compared between 3rd 2 wks and 26 wks, 4th 2 wks and 3rd 26 wks, 4th 2 wks and 3rd 2 wks, respectively. Percentage of participants with increased titers (former/later >1, orange), decreased titers (former/later <1, blue) or unchanged titers (former/later =1, green) were shown. **e** Anti-S1 (blue circle) and anti-S2 (orange triangle) antibody titers at each time point were compared. **f** Anti-RBD (blue circle) and anti-NTD (orange triangle) antibody titers at each time point were compared. **g** Antibody titers for RBD-Omicron. The orange dashed line represents the kinetics of the geometric mean titer of anti-RBD IgG. **h** The fold change of NTD- or RBD-binding IgG between 3rd 2 wks and 4th 2 wks were compared. **i** NAbs against Omicron BA.2 variants were measured by pseudovirus-based neutralization assays. **j** The fold change of NAbs against BA.1 or BA.2 from 3rd 2 wks to 4th 2 wks were compared. For (**e**–**j**), *n* = 38 biologically independent samples. Data were shown as Geometric mean ± 95% Cl in (**e**–**g**, **i**) or as box and whiskers in (**h**, **j**), indicating median (middle line), 25th, 75th percentile (box) and 5th and 95th percentile (whiskers). Two-tailed Friedman test followed by Dunn’s multiple comparisons test was used for (**e**–**g**), and two-tailed Wilcoxon matched-pairs signed rank test was used for (**h**–**j**). ns, not significant. Source data are provided as a Source Data file.

We first investigated humoral responses to S1 and S2 domains (Fig. 5a). As expected, antibody titers against these domains decreased in most participants 6 months after the 3rd dose (Fig. 5b). Both anti-S1 and anti-S2 antibodies were increased by the 4th dose but only to an inferior level as compared to that at 3rd 2 wks, suggesting antibodies to sub-domains should be taken into account (Fig. 5e). Therefore, we further measured antibodies to RBD and NTD, two pivotal sites for neutralization. Interestingly, the 4th dose had different effects on antibodies to these two sub-domains. In detail, anti-NTD titers increased in >90% of participants (Fig. 5c, 3rd bar), whereas anti-RBD titers only increased in <40% of individuals (Fig. 5c, 4th bar). The overall effect is that ~50% of participants had increased anti-NTD titers while others remained unchanged between the 3rd peak and the 4th peak (Fig. 5d, 3rd bar). In sharp contrast, >50% of participants exhibited a reduced anti-RBD titer (Fig. 5d, 4th bar), consistent with RBD-NAbs results (Fig. 4c). Both geometric mean titers of anti-RBD and anti-NTD IgG significantly dropped at 3rd 26 wks, but were boosted by the 4th dose at different slopes. Anti-NTD titers increased by 8 (5.5, 11.7) folds after the 4th dose, whereas the increase for anti-RBD was only 1.9 (1.4, 2.6) folds (Fig. 5f, 2nd and 3rd panels). Because of such a huge difference in response to the 4th dose, the ratio of anti-NTD/anti-RBD vigorously increased from 0.2 to 1.4 (Fig. 5f, 1st and 3rd panels). In another word, while anti-NTD titers were much lower than anti-RBD titers after the 3rd dose, they were boosted to a comparable or even slightly higher level over anti-RBD after receiving the 4th dose. The geometric mean titer of anti-NTD at 4th 2 wks was higher than that at 3rd 2 wks (Fig. 5f, orange triangles). In contrast, both anti-RBD and anti-RBD-Omicron titers declined from 3rd 2 wks to 4th 2 wks (Fig. 5f, g). The fold increase of NTD-specific IgGs from 3rd 2 wks to 4th 2 wks was significantly higher than that of RBD-specific IgGs (Fig. 5h).

### The 4th dose boosted NAb responses against Omicron BA.2

For WT virus, NAbs could be induced by both NTD and RBD. However, recent studies have demonstrated that most cross-neutralizing antibodies against Omicron BA.1 target majorly RBD rather than other domains in S, such as NTD^4^. BA.1 has a number of mutations and deletions in NTD, resulting in a substantial conformational change in NTD antigenic supersite^20^. A biased increment on anti-NTD rather than anti-RBD antibodies after the 4th dose was in line with our neutralizing data that the 4th dose profoundly enhanced neutralization to WT virus but not Omicron BA.1.

Whilst BA.1 and BA.2 shared similar mutations in RBD, they differed substantially in their NTD^7^. BA.2 has fewer mutations and no deletions as compared to BA.1, suggesting that anti-NTD antibodies may have a higher capability to cross-neutralize BA.2^21^. Encouragingly, the 4th dose of inactivated SARS-CoV-2 vaccines successfully elevated the peak NAbs level against BA.2 by 5.4 (2.4, 11.9) folds (Fig. 5i), in sharp contrast to BA.1, for which no enhancement was observed (Fig. 1f). The fold increase of NAbs against BA.2 from 3rd 2 wks to 4th 2 wks was significantly higher than that against BA.1 (Fig. 5j). The 4th dose was equally effective for both males and females in inducing NAbs against BA.2 (Supplementary Fig. 6). It is worth mentioning that a recent emerging Omicron XE which has a 12.6% higher growth rate above that of BA.2, shares S gene with BA.2, suggesting that the 4^th^ dose may be beneficial for strengthening the protection against both BA.2 and XE^8^.

## Discussion

In this study, we investigated the safety and effectiveness of the 4th dose of inactivated SARS-CoV-2 vaccine in HCW volunteers when a great loss of protective humoral immune responses was found for both ancestral SARS-CoV-2 and Omicron variant 6 months after the 3rd dose. At the moment, Omicron is continuously threatening the healthcare system in which HCWs are the most vulnerable population. Our results demonstrated that the inactivated SARS-CoV-2 vaccine had an acceptable safety profile that no severe side effect was found after the 4th dose despite a higher onset of adverse events (18.4%) occurring as compared to the 3rd dose (12%). The 4th dose successfully recalled the waned immune responses against both ancestral virus and Omicron variant with a low cost of safety. More importantly, the 4th dose profoundly enhanced the NAb response against Omicron BA.2, the predominant circulating VOCs at the moment. Besides this key finding, this longitudinal clinical study monitored the immune response of the same cohort for every dose, shaping a relationship between the trajectory of immune focus and the dynamics of the neutralizing potency against the evolving virus. Several implications on immunology and vaccinology are also provided by the current study.

It is a consensus that immune responses could not be endlessly boosted. A plateau or even a “turning point” would occur after repeated vaccination, but such a phenomenon has been rarely evidenced by a well-designed clinical study involving multiple administrations of the same vaccine without any interference of pre-existing immunity and asymptomatic infections during the study. Many studies as well as ours all revealed a stepwise elevation of peak immune responses from 1st to 3rd dose of SARS-CoV-2 vaccines^2,22^. No sign of a plateau or a turning point was observed before the current study enrolled a 4th dose. Our data indicated that the 3rd dose is the “turning point” for humoral responses against RBD. We observed a clear suppression of humoral response to the 4th dose by a heightened immune response after the 3rd dose. As the result of such suppression, peak levels of RBD-binding and RBD-NAbs were all inferior to their counterparts after the 3rd dose.

A recent study revealed that induction of hemagglutination inhibition (HI) antibodies, similar to RBD-NAbs in SARS-CoV-2, by a second homologous dose of influenza vaccines was attenuated by the previous dose^23^. However, the small group size of participants receiving two doses in that study (*n* = 3–8) and unknown history of natural infections prevented the study from establishing a reliable quantitative correlation. In contrast, our study enrolled a relatively large number of participants (*n* = 38) who were tested for SARS-CoV-2 weekly and had never been infected before or during the study. Thus, pre-existing immunity or occasional infection during the study which is always a concern for clinical influenza vaccine studies, would not perturb the results of this study. Taking the advantage of acceptable group size and clear immunological background, we are able to draw a clear picture that humoral immune responses to a certain region of antigens, such as RBD, were elevated dose by dose till the maximal capacity is achieved. After that, immune responses were down-regulated by a ratio tightly associate with the maximal response induced by the previous vaccination. The timing for the plateau may vary depending on the nature of antigens and adjuvants. Despite most vaccinees experiencing suppression, for those participants with a poor response to the 3rd dose, the 4th dose was still very effective.

Mechanisms underlying the down-regulation of immune responses are unclear yet. Since our data have shown that it was the peak NAb level after the 3rd dose rather than the NAb level right before the 4th dose that determined the depth of the down-regulation, we speculate that atypical memory B cells or B cell exhaustion which is always induced by repeated antigen exposure during chronic viral infection may contribute majorly, rather than other mechanisms such as epitope masking^24,25^.

Recent studies, as well as our unpublished data, revealed that the 3rd dose of inactivated vaccine or mRNA vaccine could induce a higher level of cross-neutralizing antibodies against Omicron BA.1^11,26^. Intriguingly, data in the current study indicated that the neutralization spectrum was associated with redistribution of immune responses among various epitopes after the 4th dose. Down-regulation of overheated immune responses against one domain/epitope leaves room for inducing immune responses to other epitopes, facilitating the immune system to establish a more diverse immunity which is always beneficial. However, it is not the case for the induction of cross-NAbs against Omicron BA.1. An increase of humoral immune responses to NTD domain was observed after the 4th dose accompanied by down-regulation of humoral response to RBD. However, whilst a large number of NTD-directed antibodies do neutralize WT virus, few numbers of such antibodies could cross-neutralize Omicron BA.1 since mutations induced a substantial conformational change in NTD antigenic supersite which is the target of most NTD-directed neutralizing antibodies^20^. Therefore, upregulation of NTD-induced antibodies compensated for the loss of RBD-directed neutralizing activity for WT virus but not for Omicron BA.1. Fortunately, the predominant circulating VOCs switched to BA.2 which has fewer mutations in NTD^7,21^. Our results demonstrated that the 4th dose was still effective in strengthening NAb responses against BA.2. Nevertheless, the relationship between anti-NTD antibodies and neutralization against Omicron BA.2 needs to be further studied, despite omicron neutralizing epitopes were recently found in NTD and some NTD-targeted NAbs neutralize BA.2 but not BA.1^27^.

While these results were obtained from repeated vaccination of whole inactivated SARS-CoV-2 vaccines, it will be interesting and important to know, whether down-regulation of RBD-NAbs would occur in other types of vaccines comprising RBD, and whether shifting of humoral response to other domains would also happen in vaccines comprising the whole sequence of S protein, such as mRNA-1273 and BNT162b. It is important to note, however, that widely used cross-sectional cohorts are less capable of characterizing the dynamics of neutralizing breadth and humoral responses to multiple epitopes. Instead, a longitudinal cohort, such as the cohort used in the current study, is preferred.

Our study has several limitations. First, the result came from a cohort of young HCWs. The effect of the 4th dose on very young and elderly populations may be different. Second, only a pseudovirus neutralization assay was used in the current study^16^. Nevertheless, our neutralization results regarding the ratio of NAb titers between WT/ Omicron and BA.1/BA.2 are in line with results from pseudovirus neutralization assay or authentic virus neutralization assay from other groups^11,21,22,26^.

In conclusion, our study demonstrated that the 4th dose of inactivated SARS-CoV-2 vaccine is safe and capable of further strengthening the protective immune responses against Omicron BA.2. Nevertheless, it should be noted that trajectories of the immune focus after repeated vaccinations and neutralizing epitopes of the evolving virus are not always matched. Thus, although current vaccines may still work, updated vaccines based on VOC sequences that take the advantage of RBD, NTD, and other antigenic domains would be an ideal alternative for future boosters.

## Methods

### Human subjects

In this study, we conducted a non-randomized trial and recruited participants from a prospective cohort at the First Affiliated Hospital of Sun Yat-sen University (FAH-SYSU) in Guangzhou, China. Sixty-three HCWs received a standard two-dose regimen of the inactivated SARS-CoV-2 vaccine (BBIBP-CorV, Sinopharm, Beijing) in January and February 2021^15^. Five months after the 2nd dose, 50 of the 63 HCWs volunteered to receive a 3rd dose of BBIBP-CorV in July 2021, just before the global circulation of Delta VOC^2^. Thirty-eight of them volunteered for the current study aiming to investigate the safety and effectiveness of a 4th dose, at the moment when healthcare systems were challenged by the Omicron variant and the immune response induced by the 3rd vaccination waned substantially. Although dropping out randomly, this population has a similar age distribution (median, IQR) of 25 (24, 29) by the end of 2021 to the two-dose cohort 26 (24, 28) and third-dose cohort 26 (24, 28), while containing more males (47.4%) than the previous cohorts (41.3% and 40%). Moreover, this population has a similar baseline of RBD-NAbs to the original cohort (63 participants) at 2nd 4 wks, and the cohort participating in the 3rd-dose trial (50 participants) at 3rd 2 wks (Supplementary Fig. 7). They received a 4th homologous booster shot of the inactivated vaccine in January 2022, 6 months after the 3rd vaccination. Blood samples were collected right before the booster dose, 14 days, 28 days, and 3 months after the booster. Convalescent patients who had recovered from WT SARS-CoV-2 infection were recruited as the positive control for ADCC assay. The convalescent sera were collected 407.6 (403-411) days post-diagnostic^2^. All studies were approved by IEC for Clinical Research and Animal Trials of the First Affiliated Hospital of Sun Yat-sen University and written consent was obtained from all participants. Participants received compensation for attending this study. The prospective cohort and the trial were registered to the Chinese Clinical Trial Registry (ChiCTR2100042222, ChiCTR2200055564).

### Blood samples

For serum collection, blood samples were allowed to clot at room temperature and subsequently centrifuged at 3000 × *g* for 10 min. Sera were transferred into 0.5 ml aliquots in polypropylene tubes and stored at −80 °C. To isolate peripheral blood mononuclear cells (PBMCs), blood samples were collected into the heparinized tubes. PBMCs were isolated by density-gradient centrifugation. Briefly, blood samples were diluted with PBS at a 1:1 ratio to 30 ml and loaded on top of 15 ml Lymphoprep™ (StemCell) in the 50 ml centrifugation tube and centrifuged at 700 × *g* for 30 min. The medium cell layer was collected and washed with PBS once, followed by centrifugation at 245 × *g* for 10 min. Pelleted PBMCs were cryopreserved in Bambanker (StemCell) immediately at −80 °C.

### Cell lines and plasmids

Human ACE2 over-express HEK293T (hACE2-293T, PackGene Biotech) were cultured in DMEM (10-013-CVRC, Corning) supplemented with 10% fetal bovine serum (FBS, FSP500, ExCellBio), non-essential amino acids (NEAA, 11140-050, Gibco), 100 U/ml penicillin and 100 μg/ml streptomycin (SV30010, HyClone). Jurkat-Lucia™ NFAT-CD16 Cells (jktl-nfat-cd16, InvivoGen) were cultured in IMDM (BL312A, Biosharp) supplemented with 10% FBS, NEAA, 100 U/ml penicillin and 100 μg/ml streptomycin, 100 μg/ml Zeocin (ST-1450, Beyotime) and 10 μg/ml Blasticidin S (ST-018, Beyotime). All cell lines were passaged less than 15 generations and examined the mycoplasma by PCR and fluorescence labeling methods.

The plasmid pcDNA3.1-2019-nCoV-Spike is a gift from Dr. Lu Lu at Fudan University, encoding the spike protein from an ancestral SARS-CoV-2 reference strain (Wuhan-Hu-1) which is called as wild type (WT) throughout the manuscript. The pcDNA3.1(+) plasmids encoding the spike protein from the Omicron variant (B1.1.529) BA.1 or BA.2 lineage were synthesized by Kidan Bio. The plasmid pcDNA3.1(+)-Envelope encodes envelope protein from WT was full-genome synthesized by Genewiz China according to the reference sequence NC_045512.2 in NCBI. Plasmids pSPAX2 and pLenti-CMV-Puro-Luc (168w-1) were a gift from Dr. Jianping Guo and purchased from MiaolingBio (P1216), respectively.

### ELISA

All SARS-CoV2 recombinant proteins were purchased from Sino Biological (Beijing, China). For ELISA, 200 ng/well of WT SARS-CoV2 spike (40589-V08B1), spike S1 subunit (40591-V08H), spike S2 subunit (40590-V08B), NTD (40591-V49H), RBD (40592-V08H), nucleocapsid (40588-V08B) and envelope (40609-V10E3), Omicron spike (40589-V08H26), spike S1 subunit (40591-V08H41), RBD (40592-V08H121) and nucleocapsid (40588-V07E34) were coated on the 96-well ELISA plate (655061, Greiner Bio-one) using coating buffer (G3022, Saint Bio) overnight at 4 °C, respectively. Plates were washed by PBS supplemented with 0.5% Tween-20 (PBST) for three times, followed by blocking with 5% BSA in PBST (blocking buffer) for 1 h at room temperature. Sera were firstly diluted 40-fold, followed by 4-fold serial dilution and incubation at 4 °C overnight. Plates were washed 5 times by PBST, and incubated with 100 μl/well goat HRP conjugated anti-human IgG antibody (2040-05, SouthernBiotech, 1:3000) in PBST at room temperature for 30 min. After washing 5 times with PBST, 100 μl/well 3,3′,5,5′-Tetramethylbenzidine substrate (P0209, Beyotime) was added to each well for 15 min, stopped by the stopping buffer (P0215, Beyotime). OD450 was measured by Varioskan Lux Microplate Reader (Thermo Fisher).

### Pseudovirus neutralization assay

Pseudovirus production and neutralization assay were performed following a previous study^16^. To generate WT SARS-CoV-2-Spike (Wuhan-Hu-1) pseudovirus, pcDNA3.1-2019-nCoV-Spike, pSPAX2 and pLenti-CMV-puro-Luc (168w-1) were co-transfected to HEK293T (maintained in the laboratory) using Lipo8000 (C0533, Beyotime) according to the manufacturer’s instruction. For the generation of the B.1.1.529 Omicron-variant spike pseudovirus, pcDNA3.1(+)-Omicron-spike, pSPAX2 and pLenti-CMV-puro-Luc (168w-1) were co-transfected to HEK293T using Lipo8000. The virus-containing supernatant was harvested after 72 h and stored at −80 °C until use. The hACE2-293T at 2 × 10^4^/well were seeded on the black flat-bottom 96-well plate (655090, Greiner Bio-one) for 16 h in advance. Sera were firstly diluted 10-fold then 4-fold serial diluted subsequently in DMEM, then co-incubated with pseudovirus at 37 °C for 1 h. The co-incubated samples, together with samples without sera or pseudovirus as controls, were subjected with 10 μg/ml polybrene (C0351, Beyotime) to the hACE2-293T for 6-h absorption. The culture medium was replaced and incubated for another 42 h at 37 °C. Infected cells were lysed by firefly luciferase lysis buffer (RG126M, Beyotime), then the luciferase substrate (RG058M, Beyotime) was applied for the luciferase assay according to the manufacturer’s instruction. The relative light unit (RLU) was measured by Varioskan Lux Microplate Reader (Thermo Fisher). The 50% pseudovirus neutralization titer (PVNT50) was determined by a four-parameter nonlinear regression curve (GraphPad Prism).

### Neutralizing antibodies against RBD

A one-step competitive Chemiluminescent immunoassay was used to detect the concentration of NAbs against RBD (RBD-NAbs) in sera by iFlash 2019-nCoV NAb kit (C86109, YHLO Biotech Co, Ltd)^2^. Briefly, the RBD of the SARS-CoV-2 was coated on magnetic beads. Acridinium ester-labeled ACE2 protein was used to compete with serum NAbs for the RBD. Titers of RBD-NAbs were calculated by an iFlash3000 Chemiluminescence Immunoassay Analyzer (YHLO Biotech Co, Ltd). Neutralizing activity is determined in arbitrary units (AU) and the cut-off is 10 AU/ml.

### ELISpot

The PBMCs were thawed, resuspended in 10% FBS RPMI1640 and stained with trypan blue (Meilunbio, MA0130) for the dead cell exclusion. The stained cell suspension was then counted by an automated cell counter (Model R1, Olympus). 3 × 10^5^ live cells/well were seeded on the human IFNγ pre-coated ELISpot kit (Dakewe, 2110006) plates and allowed to rest for 2 h at 37 °C. Then the cells were cultured with 2 μg/ml peptide pool of SARS-CoV-2 spike protein (PP003, Sino Biological) or nucleocapsid^28^ in the presence of 2 μg/ml of anti-human CD28 monoclonal antibody (302934, Clone CD28.2, Biolegend) for 24 h. Unstimulated cells were used as negative control while 5 μg/ml anti-human CD3 (317326, Clone OKT3, Biolegend) and 2 μg/ml of anti-human CD28 monoclonal antibodies stimulated cells were used as positive control. Plates were treated according to the manufacturer’s instruction. In brief, the plates were incubated with the biotinylated anti-human IFNγ antibody (2110006, Clone 1-D1K, 1:100), followed by incubation with streptavidin-HRP and the ACE staining. The plates were then scanned and the spots were counted by ImmunoSpot Analyser (Cellular Technology Ltd.).

### ADCC assay

The luciferase-based ADCC assay was performed following a previous study^18^. To generate antigen-expressing target cells, HEK293T cells were transfected with pcDNA3.1-2019-nCoV-Spike, pcDNA3.1(+)-Omicron-spike or pcDNA3.1(+)-Envelope. Target cells were harvested 48 h post-transfection and seeded at 4 × 10^4^/well on the black flat-bottom 96-well plates for 10 h. Sera were diluted 10-fold for the first well and 4-fold serial diluted for subsequent wells in DMEM, then were incubated with the target cells for 1 h at 37 °C. Cells were washed by warm culture medium once, followed by adding 8 × 10^4^/well Jurkat-Lucia™ NFAT-CD16 Cells (InvivoGen). Twelve hours later, 50 μl reconstructed Quanti-Luc (rep-qlc2, InvivoGen) was added to each well. The luminescence was measured immediately by Varioskan Lux Microplate Reader. The ADCC activity was quantified as fold induction. The area under the curve (AUC) was first calculated by plotting RLU (serum sample–no serum control) against log(plasma dilution). Then, ADCC fold induction was calculated as AUC-vaccinee/AUC-unimmunized control.

### Statistical analysis

The main objective of this study is to explore the impact of the 4th dose of inactivated SARS-CoV-2 vaccine on recalling waned immune responses against WT virus and VOCs. Most analyses were designed in a pre-specified manner, including longitudinal comparisons of NAbs against WT and Omicron BA.1, RBD-NAbs, ADCC activities, T cell responses, and ELISA titers at different time points. Some analyses were taken in a post-hoc manner, including all correlation analyses, comparison between humoral responses to different antigens, and NAbs against Omicron BA.2 which was not the major circulating strain when the study was designed. All analyses included all 38 participants who were in our prospective cohort and volunteered to receive the 4th dose of inactivated vaccines. All samples were collected within 2 days at each indicated time point. All blood samples are available for every participant at every time point. Statistical analysis was performed using Graphpad Prism 6. Data were shown as Geometric mean ± 95% Cl unless indicated otherwise. Comparisons were assessed using Wilcoxon matched-pairs signed-rank test, paired Student’s *t* test, Friedman test followed by Dunn’s multiple comparisons test, or RM ANOVA followed by Bonferroni’s multiple comparisons test. *P* values <0.05 were considered as statistically significant.

### Reporting summary

Further information on research design is available in the Nature Portfolio Reporting Summary linked to this article.

## Supplementary information

Supplementary Information

Reporting Summary

## Data Availability

All data supporting the findings of this study are available within the paper and supplementary materials. Source data are provided with this paper. The other individual de-identified participant data could be shared by the corresponding author upon reasonable request. The study protocol is available as a supplementary file. Source data are provided with this paper.

## Source data

Source Data

## References

[1] Levin EG (2021). Waning immune humoral response to BNT162b2 Covid-19 vaccine over 6 months. N. Engl. J. Med..

[2] Liu Y (2022). Robust induction of B cell and T cell responses by a third dose of inactivated SARS-CoV-2 vaccine. Cell Discov..

[3] Wu F (2020). A new coronavirus associated with human respiratory disease in China. Nature.

[4] Cao, Y. et al. Omicron escapes the majority of existing SARS-CoV-2 neutralizing antibodies. *Nature* **602**, 657–663 (2021). 10.1038/s41586-021-04385-3 PMC8866119 35016194

[5] Falsey AR (2021). SARS-CoV-2 neutralization with BNT162b2 vaccine dose 3. N. Engl. J. Med..

[6] Kuhlmann C (2022). Breakthrough infections with SARS-CoV-2 omicron despite mRNA vaccine booster dose. Lancet.

[7] Stalls V (2022). Cryo-EM structures of SARS-CoV-2 Omicron BA.2 spike. Cell Rep..

[8] Agency, U.H.S. SARS-CoV-2 Variants of Concern and Variants under Investigation in England, Technical briefing 40 (2022).

[9] Khan, K. et al. Omicron sub-lineages BA.4/BA.5 escape BA.1 infection elicited neutralizing immunity. *medRxiv*10.1101/2022.04.29.22274477 (2022).

[10] Fireman, J.M.F.S.R.B.E.D.P.K.M.M.B.D.B. Waning 2-Dose and 3-Dose Effectiveness of mRNA Vaccines Against COVID-19—Associated Emergency Department and Urgent Care Encounters and Hospitalizations Among Adults During Periods of Delta and Omicron Variant Predominance—VISION Network, 10 States, August 2021–January 2022. *Morbidity and Mortality Weekly Report* 71 (2022). 10.15585/mmwr.mm7107e2 PMC8853475 35176007

[11] Muik A (2022). Neutralization of SARS-CoV-2 Omicron by BNT162b2 mRNA vaccine-elicited human sera. Science.

[12] Keeton, R. et al. T cell responses to SARS-CoV-2 spike cross-recognize Omicron. *Nature* 604, E25 (2022). 10.1038/s41586-022-04708-y PMC8993033 35396582

[13] Bar-On, Y.M. et al. Protection by 4th dose of BNT162b2 against Omicron in Israel. *N. Engl. J. Med.* **386**, 1712–1720 (2022). 10.1056/NEJMoa2201570 PMC9006780 35381126

[14] Regev-Yochay, G. et al. 4th Dose COVID mRNA Vaccines’ Immunogenicity & Efficacy Against Omicron VOC. *MedRxiv*10.1101/2022.02.15.22270948 (2022).

[15] Zhang H (2021). Time of day influences immune response to an inactivated vaccine against SARS-CoV-2. Cell Res..

[16] Tong P (2021). Memory B cell repertoire for recognition of evolving SARS-CoV-2 spike. Cell.

[17] Dugan HL (2021). Profiling B cell immunodominance after SARS-CoV-2 infection reveals antibody evolution to non-neutralizing viral targets. Immunity.

[18] Keeton R (2021). Prior infection with SARS-CoV-2 boosts and broadens Ad26.COV2.S immunogenicity in a variant-dependent manner. Cell Host Microbe.

[19] Tenbusch M (2021). Heterologous prime-boost vaccination with ChAdOx1 nCoV-19 and BNT162b2. Lancet Infect. Dis..

[20] Gabriele Cerutti, Y.G. et al. Cryo-EM structure of the SARS-CoV-2 omicron spike. *Cell Rep*. 38, 110428 (2022). 10.1016/j.celrep.2022.110428 PMC8818377 35172173

[21] Yu J (2022). Neutralization of the SARS-CoV-2 Omicron BA.1 and BA.2 Variants. N. Engl. J. Med..

[22] Wang, K. et al. Memory B cell repertoire from triple vaccinees against diverse SARS-CoV-2 variants. *Nature* **603**, 919–925 (2022). 10.1038/s41586-022-04466-x PMC8967717 35090164

[23] Khurana S (2019). Repeat vaccination reduces antibody affinity maturation across different influenza vaccine platforms in humans. Nat. Commun..

[24] Bergstrom JJ, Xu H, Heyman B (2017). Epitope-specific suppression of igg responses by passively administered specific IgG: evidence of epitope masking. Front. Immunol..

[25] Moir S, Fauci AS (2014). B-cell exhaustion in HIV infection: the role of immune activation. Curr. Opin. HIV AIDS.

[26] Garcia-Beltran WF (2022). mRNA-based COVID-19 vaccine boosters induce neutralizing immunity against SARS-CoV-2 Omicron variant. Cell.

[27] Wang, Z. et al. Conserved neutralizing epitopes on the N-terminal domain of variant SARS-CoV-2 spike proteins. *bioRxiv*10.1101/2022.02.01.478695 (2022). 10.1016/j.immuni.2022.04.003 PMC8986478 35447092

[28] Peng Y (2020). Broad and strong memory CD4(+) and CD8(+) T cells induced by SARS-CoV-2 in UK convalescent individuals following COVID-19. Nat. Immunol..

