# Supplementary material for: A fourth dose of the inactivated SARS-CoV-2 vaccine redistributes humoral immunity to the N-terminal domain
Source: Nat Commun. 2022 Nov 11;13:6866. doi: 10.1038/s41467-022-34633-7 (PMC9651894; doi:10.1038/s41467-022-34633-7)
Supplement: Supplementary file 1 — Supplementary Information [file 41467_2022_34633_MOESM1_ESM.pdf]

Supplementary Information

A fourth dose of the inactivated SARS-CoV-2 vaccine redistributes humoral immunity to the N-terminal domain

Ji Wang[1,2#*], Caiguangxi Deng[2#], Ming Liu[2#], Yihao Liu[3#], Liubing Li[4], Zhangping Huang[2], Liru Shang[2], Juan Jiang[2], Yongyong Li[2], Ruohui Mo[3], Hui Zhang[2,5], Min Liu[4], Sui Peng[2,3*], Haipeng Xiao[1*&]

[#]These authors contributed equally to this work.

*Correspondence to Dr. Haipeng Xiao, xiaohp@mail.sysu.edu.cn; Dr. Sui Peng, pengsui@mail.sysu.edu.cn; Dr. Ji Wang, wangj683@mail.sysu.edu.cn

[&] Lead contact

Supplementary Fig. 1-7

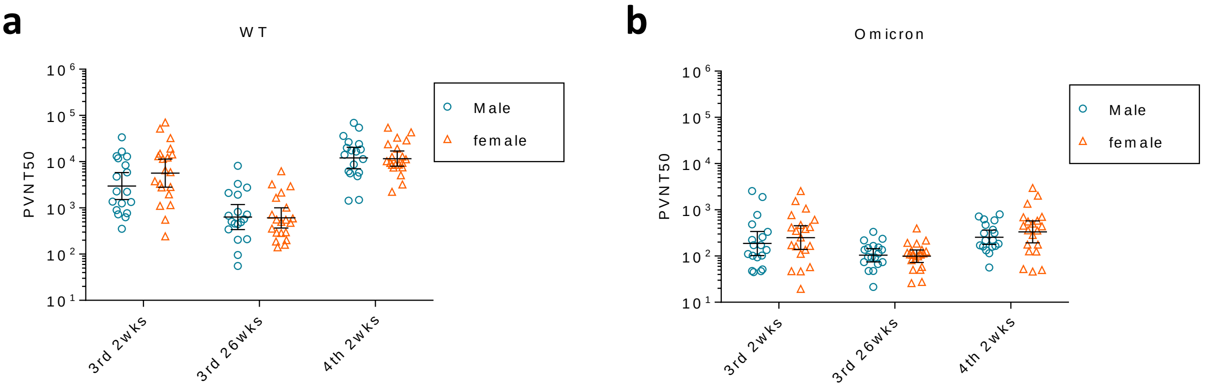

Supplementary Figure 1 NAbs in males and females

Neutralization assays were performed to measure NAbs titers against pseudoviruses with S protein from a WT strain or Omicron variant. (**a**) NAb titers for WT virus in males (blue circle) or females (orange triangle) were compared. (**b**) NAb titers for Omicron variant were similarly compared between males (blue circle) or females (orange triangle). For male group, n=18 biologically independent samples. For female group, n=20 biologically independent samples. Data were shown as Geometric mean ± 95% Cl. Source data are provided as a Source Data file.

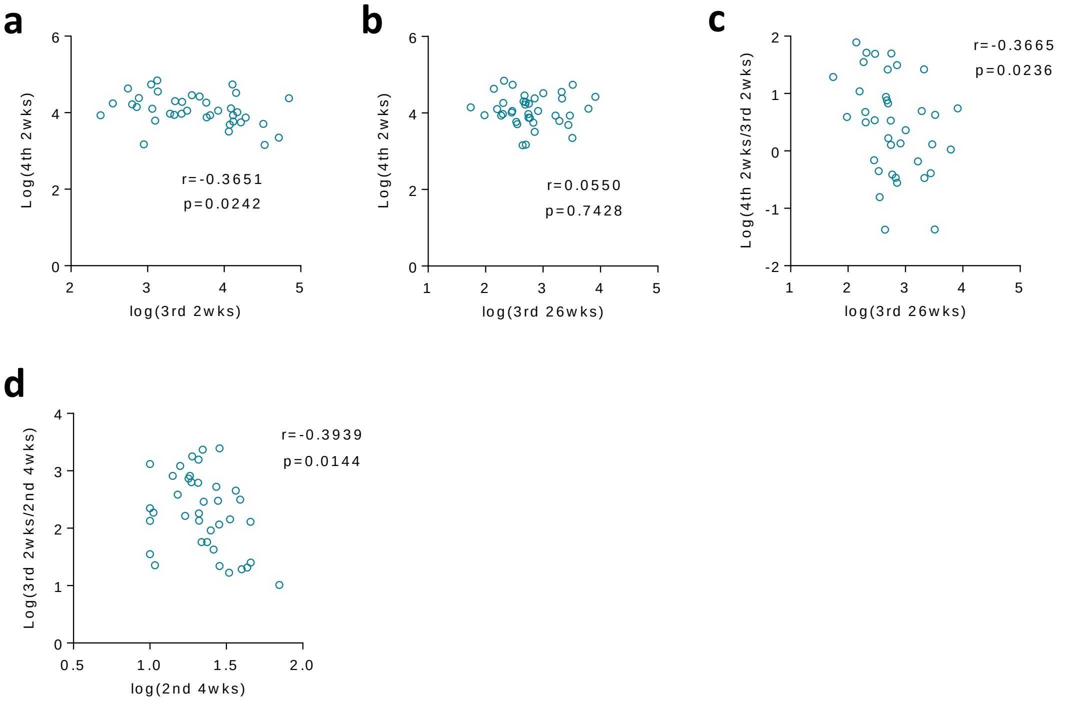

Supplementary Figure 2 Correlation between NAb levels at various time points

(**a-c**) The two-tailed spearman correlation coefficient was calculated for NAb levels at 3rd 2wks and 4th 2wks (**a**), or NAb levels between 3rd 26wks and 4th 2wks (**b**), or fold change of peak values and NAb level at 3rd 26wks (**c**). (**d**) The two-tailed spearman correlation coefficient was calculated for the fold-change of PVNT50 from 2nd 4wks to 3rd 2wks and PVNT50 at 2nd 4wks. Source data are provided as a Source Data file.

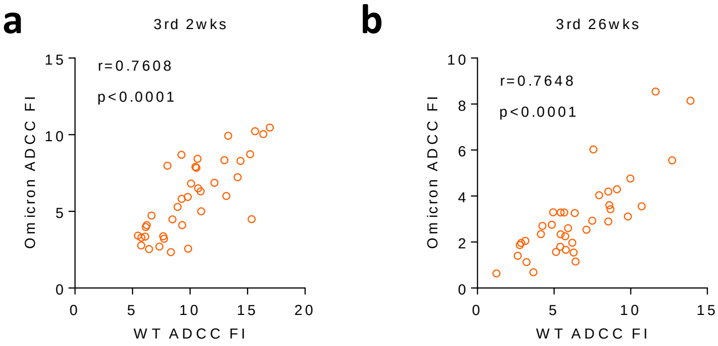

Supplementary Figure 3 ADCC activity against Omicron was tightly correlated with that against WT

The two-tailed spearman correlation coefficient was calculated for ADCC activities against WT and Omicron at 3rd 2wks (**a**) or 3rd 26wks (**b**). Source data are provided as a Source Data file.

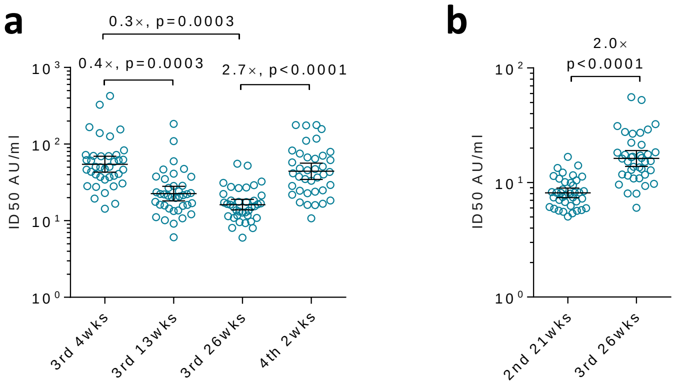

Supplementary Figure 4 Comparison of RBD-NAbs at various time points

RBD-NAbs were measured by a one-step competitive Chemiluminescent immunoassay. (**a**) RBD-NAbs at 3rd 4wks, 13wks, 26wks and 4th 2wks were compared. (**b**) RBD-NAbs at 2nd 21wks and 3rd 26wks were compared. n=38 biologically independent samples. Data were shown as Geometric mean ± 95% Cl. RM one-way ANOVA followed by Bonferroni's multiple comparisons test was used for (**a**), and two-tailed paired t-test was used for (**b**). Source data are provided as a Source Data file.

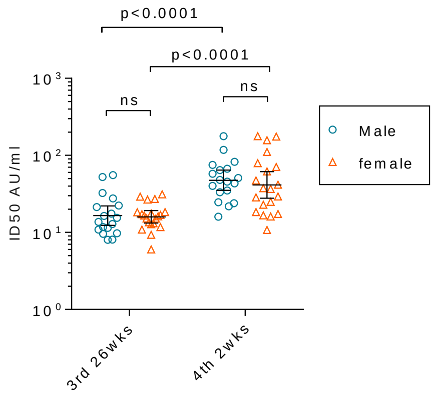

Supplementary Figure 5 RBD-NAbs in males and females

RBD-NAbs were measured by a one-step competitive Chemiluminescent immunoassay. RBD-NAb titers in males (blue circle) or females (orange triangle) at 3rd 26wks or 4th 2wks were compared. For male group, n=18 biologically independent samples. For female group, n=20 biologically independent samples. Data were shown as Geometric mean ± 95% Cl. Two-tailed Mann-Whitney test was used for comparison between males and females, and two-tailed Wilcoxon matched-pairs signed rank test was used for comparison between different time points. ns, not significant. Source data are provided as a Source Data file.

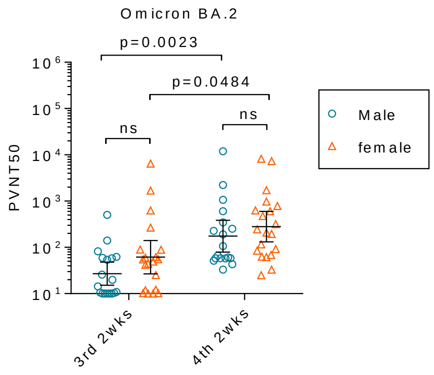

Supplementary Figure 6 NAbs against Omicron BA.2 in males and females

Neutralization assays were performed to measure NAbs titers against pseudoviruses with S protein from an Omicron BA.2 variant. NAb titers in males (blue circle) or females (orange triangle) were compared. For male group, n=18 biologically independent samples. For female group, n=20 biologically independent samples. Data were shown as Geometric mean ± 95% Cl. Two-tailed Mann-Whitney test was used for comparison between males and females, and two-tailed Wilcoxon matched-pairs signed rank test was used for comparison between different time points. ns, not significant. Source data are provided as a Source Data file.

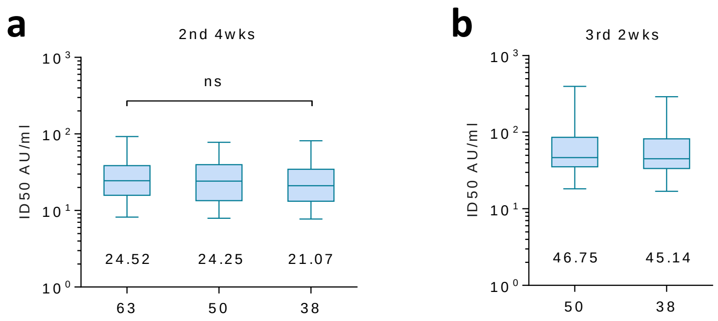

Supplementary Figure 7 Comparison of RBD-NAbs among three cohorts

RBD-NAbs were measured by a one-step competitive Chemiluminescent immunoassay. (**a**) RBD-NAbs at 2nd 4wks of all volunteers participating in the two-dose trial (63), or the subgroup participating in the 3rd-dose trial (50), or the subgroup participating in the 4th-dose trial (38) were compared. (**b**) RBD-NAbs at 3rd 2wks were compared. Data were shown as box and whiskers, indicating median (middle line and the number), 25th, 75th percentile (box) and 5th and 95th percentile (whiskers). One-way ANOVA followed by Bonferroni's multiple comparisons test was used for (**a**). ns, not significant. Source data are provided as a Source Data file.
