# Supplementary material for: A fourth dose of the inactivated SARS-CoV-2 vaccine redistributes humoral immunity to the N-terminal domain
Source: Nat Commun. 2022 Nov 11;13:6866. doi: 10.1038/s41467-022-34633-7 (PMC9651894; doi:10.1038/s41467-022-34633-7)
Supplement: Supplementary file 2 — Reporting Summary [file 41467_2022_34633_MOESM2_ESM.pdf]

nature portfolio

Corresponding author(s): Haipeng Xiao, Sui Peng, Ji Wang

Last updated by author(s): Oct 31, 2022

Reporting Summary

Nature Portfolio wishes to improve the reproducibility of the work that we publish. This form provides structure for consistency and transparency in reporting. For further information on Nature Portfolio policies, see our Editorial Policies and the Editorial Policy Checklist.

Statistics

For all statistical analyses, confirm that the following items are present in the figure legend, table legend, main text, or Methods section.

n/a	Confirmed	
☐	☒	The exact sample size (*n*) for each experimental group/condition, given as a discrete number and unit of measurement
☐	☒	A statement on whether measurements were taken from distinct samples or whether the same sample was measured repeatedly
☐	☒	The statistical test(s) used AND whether they are one- or two-sided<br>*Only common tests should be described solely by name; describe more complex techniques in the Methods section.*
☐	☒	A description of all covariates tested
☐	☒	A description of any assumptions or corrections, such as tests of normality and adjustment for multiple comparisons
☐	☒	A full description of the statistical parameters including central tendency (e.g. means) or other basic estimates (e.g. regression coefficient) AND variation (e.g. standard deviation) or associated estimates of uncertainty (e.g. confidence intervals)
☐	☒	For null hypothesis testing, the test statistic (e.g. *F*, *t*, *r*) with confidence intervals, effect sizes, degrees of freedom and *P* value noted<br>*Give P values as exact values whenever suitable.*
☐	☒	For Bayesian analysis, information on the choice of priors and Markov chain Monte Carlo settings
☐	☒	For hierarchical and complex designs, identification of the appropriate level for tests and full reporting of outcomes
☐	☒	Estimates of effect sizes (e.g. Cohen's *d*, Pearson's *r*), indicating how they were calculated

Our web collection on statistics for biologists contains articles on many of the points above.

Software and code

Policy information about availability of computer code

Data collection	No software was used for data collection
Data analysis	Graphpad Prism 6

For manuscripts utilizing custom algorithms or software that are central to the research but not yet described in published literature, software must be made available to editors and reviewers. We strongly encourage code deposition in a community repository (e.g. GitHub). See the Nature Portfolio guidelines for submitting code & software for further information.

Data

Policy information about availability of data

All manuscripts must include a data availability statement. This statement should provide the following information, where applicable:

- Accession codes, unique identifiers, or web links for publicly available datasets
- A description of any restrictions on data availability
- For clinical datasets or third party data, please ensure that the statement adheres to our policy

All data supporting the findings of this study are available within the paper and supplementary materials. Source data are provided with this paper.
The other individual de-identified participant data could be shared by the corresponding author upon reasonable request. The study protocol is available as a supplementary file.

Field-specific reporting

Please select the one below that is the best fit for your research. If you are not sure, read the appropriate sections before making your selection.

☒ Life sciences ☐ Behavioural & social sciences ☐ Ecological, evolutionary & environmental sciences

For a reference copy of the document with all sections, see nature.com/documents/nr-reporting-summary-flat.pdf

Life sciences study design

All studies must disclose on these points even when the disclosure is negative.

Sample size	As a pilot study, no sample size calculation was performed in this work. We recruited all possible participants from our previous prospective cohort.
Data exclusions	No data was excluded.
Replication	All experiments were performed at least twice with similar results.
Randomization	This a non-randomized clinical study. We recruited all possible participants from our previous prospective cohort.
Blinding	All experiments were performed and analyzed in an investigator-blind fashion.

Reporting for specific materials, systems and methods

We require information from authors about some types of materials, experimental systems and methods used in many studies. Here, indicate whether each material, system or method listed is relevant to your study. If you are not sure if a list item applies to your research, read the appropriate section before selecting a response.

Materials & experimental systems

n/a	Involved in the study	
☐	☒	Antibodies
☐	☒	Eukaryotic cell lines
☒	☐	Palaeontology and archaeology
☒	☐	Animals and other organisms
☐	☒	Human research participants
☐	☒	Clinical data
☒	☐	Dual use research of concern

Methods

n/a	Involved in the study	
☒	☐	ChIP-seq
☒	☐	Flow cytometry
☒	☐	MRI-based neuroimaging

Antibodies

Antibodies used	Goat HRP conjugated anti-human IgG (2040-05, SouthernBiotech, 1:3000), anti-human CD28 monoclonal antibody (302934, Clone CD28.2, Biolegend, 2 μg/ml), anti-human CD3 (317326, Clone OKT3, Biolegend, 5 μg/ml), biotinylated anti-human IFNγ antibody (2110006, Clone 1-D1K, Lot. 2202-1, 1:100)
Validation	All antibodies have been validated by vendors for the species and applications. Validation statements could be found on the manufacturer's website. Negative and Positive controls were used to further validate the application in each experiment.

Eukaryotic cell lines

Policy information about cell lines

Cell line source(s)	Human ACE2 over-express HEK293T (hACE2-293T, PackGene Biotech), Jurkat-Lucia™ NFAT-CD16 Cells (jktl-nfat-cd16, InvivoGen). For cell lines, sex was not considered in the study design.
Authentication	Human ACE2 over-express HEK293T was purchased from PackGene Biotech (China) Inc. The manufacturer has provided the STR profiling to authenticate the the cell line as HEK293T, together with qPCR, Western Blot and GFP-pseudotype SARS-CoV-2 virus to validate the expression of human ACE2 receptor. The Jurkat-Lucia™ NFAT-CD16 Cells was purchased from the InvivoGen Inc. The company has provided a certificate to declare the cell line was authenticated as Jurkat by STR profiling, guarantee the overexpression of human CD16 receptor by flow cytometry. NFAT transcriptional factor and Lucia secretory luciferase was determined by the functional assay.
Mycoplasma contamination	All cell lines were tested negative for the mycoplasma contamination by PCR and fluorescence labeling methods.

Commonly misidentified lines (See ICLAC register)

No commonly misidentified cell lines were used in the study.

Human research participants

Policy information about studies involving human research participants

Population characteristics	median,IQR 25 24 29 , male 47.4%
Recruitment	We recruited all possible participants from our previous prospective cohort.<br>1. participants aged 18-59 years old;<br>2. participants who had finished the third dose of inactivated vaccine against SARS-CoV-2 ((BBIBP-CorV) more than 6 months.<br>3. Sex was not considered in the study design. Sex of participants was determined based on self-report.
Ethics oversight	IEC for Clinical Research and Animal Trials of the First Affiliated Hospital of Sun Yat-sen University

Note that full information on the approval of the study protocol must also be provided in the manuscript.

Clinical data

Policy information about clinical studies

All manuscripts should comply with the ICMJE guidelines for publication of clinical research and a completed CONSORT checklist must be included with all submissions.

Clinical trial registration	ChiCTR2100042222, ChiCTR2200055564
Study protocol	The study protocol is available as a supplementary file.
Data collection	We recruited participants from our previous prospective cohort at the First Affiliated Hospital of Sun Yat-sen University (FAH-SYSU) in January 2022. Blood samples were collected right before the booster dose, 14 days, 28 days, and 3 months after the vaccination at FAH-SYSU.
Outcomes	The primary (Neutralizing antibodies) and secondary outcomes (RBD antibodies) were pre-defined since they were reported to play pivotal roles in the SARS-CoV-2 vaccine-induced immune protection. Neutralizing antibodies were measured by pseudotype virus neutralization assay. RBD antibodies were measured by a one-step competitive Chemiluminescent immunoassay and ELISA. T cell responses were measured by ELISpot. Adverse events were defined as any adverse event occurred during the time when the participants enter the trial to the last visit, regardless of whether there is a causal relationship with the inactivated SARS-CoV-2 vaccine. Adverse events were documented according to the Common Terminology Criteria for Adverse Events (CTCAE), version 5.0.
